# Not only self-views but also any ratings converge without conversations due to reduced noise bias

**DOI:** 10.1073/pnas.2505968122

**Published:** 2025-06-20

**Authors:** Keise Izuma, Kyosuke Kakinuma

**Affiliations:** ^a^School of Economics and Management, Kochi University of Technology, Kochi 780-8515, Japan; ^b^Research Center for Mind, Brain, and Behavior, Kochi University of Technology, Kochi 780-8515, Japan; ^c^School of Psychology, University of Southampton, Southampton SO17 1BJ, United Kingdom; ^d^Japan Society for the Promotion of Science, Tokyo 102-0083, Japan

A recent study by Welker et al. (1) found that people’s self-views tend to become more similar after just a 10-min conversation, a process the authors termed “inter-self alignment (ISA).” However, the study lacked a control condition (e.g., a no-conversation condition), leaving open the possibility that the observed ISA could occur without any social interaction.

**Fig. 1.**
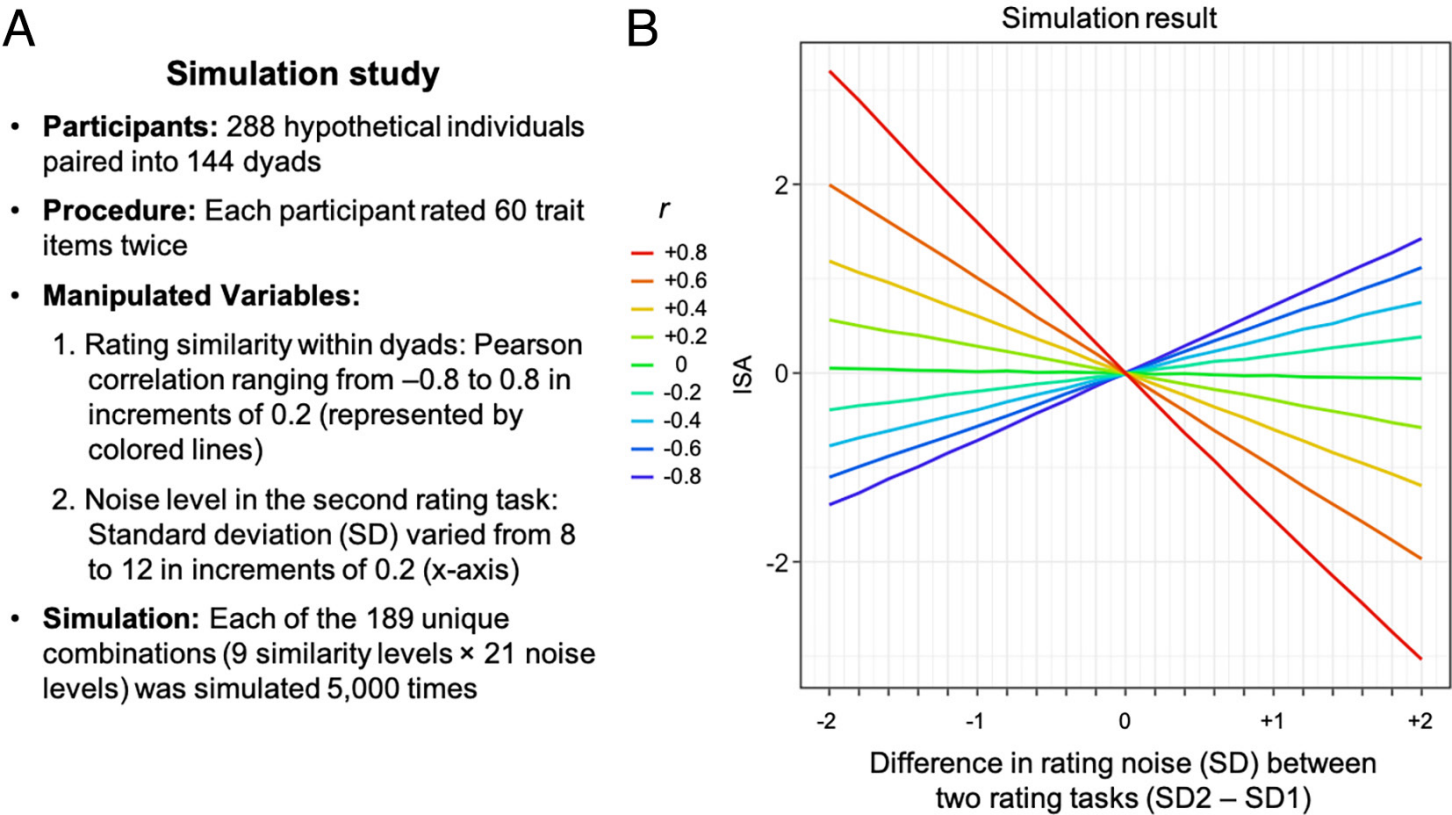
The simulation study. (*A*) Within each pair, one participant’s “true self-view” scores were generated by randomly drawing 60 values from a normal distribution (mean = 50, SD = 18). The other participant’s self-view scores were randomly generated to correlate with their partner’s self-view at one of nine levels. We added random noise to each true self-view score to generate a “temporal self-view” score. In the first rating task (baseline), noise was drawn from a normal distribution (SD = 10). For the second rating task, noise levels varied systematically (21 levels), while true self-views remained unchanged. The resulting temporal self-view scores were rounded to the nearest whole number, serving as the “observed ratings” used to compute ISA. (*B*) The results showed positive ISA when i) noise is smaller in the second rating task and ii) participant’s ratings were positively correlated (upper-left quadrant) (and when neither condition is met). The simulation code is available here (https://osf.io/948js/).

Here, through simulations and empirical studies, we demonstrate that significant ISA can arise solely due to a statistical artifact—specifically, a reduction in rating noise across successive rating sessions within a short interval. Our simulation (Fig. 1) showed that even when true self-views remain unchanged, ISA can emerge under two conditions: i) rating noise decreases over successive rating tasks, and ii) participants’ ratings are positively correlated. Notably, we directly communicated with the authors (1) and confirmed that both assumptions held in the original data.

**Fig. 2.**
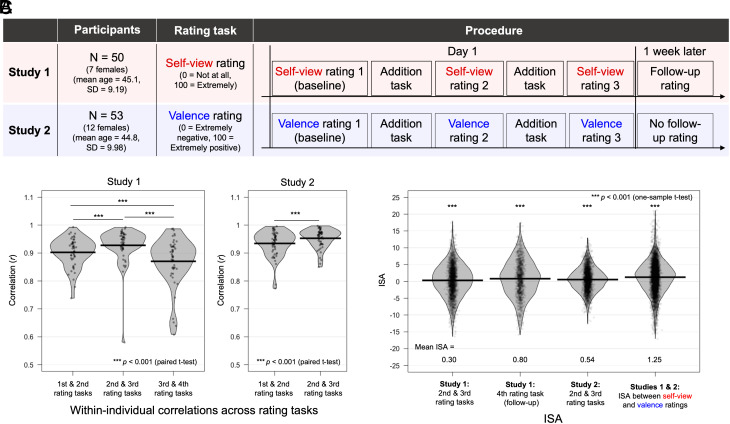
Procedures and results of the two online studies. (*A*) Study design. In both studies, participants rated the same 60 traits (1). The order of the 60 traits was randomized in each rating task. Each rating task was separated by an arithmetic addition task (~10 min). Participants were explicitly instructed not to communicate with anyone during the study. ISA scores were computed following the method used in the original study for all possible participant pairs. (*B*) Correlations between successive rating sessions. To examine whether rating noise decreased across sessions, we computed item-by-item correlations within each individual. If noise is reduced, successive ratings should show higher correlations. The results revealed that correlations increased across rating sessions on the first day, indicating that rating noise decreased over time (and increased again after a week). (*C*) ISA bias. ISA was significantly biased in the positive direction regardless of rating types and even when ratings were on different dimensions. The dataset is available here (https://osf.io/948js/). Both studies were approved by the Kochi University of Technology Ethics Committee, and all participants provided informed consent before completing the questionnaire.

Our simulations suggest that ISA can occur without conversation and for any type of ratings, as long as these two conditions are met. To test this, we conducted two online studies: self-view ratings in Study 1 and valence ratings in Study 2 (Fig. 2*A*). Across both studies, item-by-item correlations indicated that rating noise decreased across successive rating tasks conducted within a short time frame but increased after a week (Fig. 2*B*). Additionally, participants’ baseline ratings were positively correlated (Study 1: mean *r* = 0.27; Study 2: mean *r* = 0.85, both *Ps* < 0.001), confirming that both conditions necessary for ISA were met.

As predicted, we found significant ISA, including in the follow-up session (Fig. 2*C*). Thus, ISA can occur without any conversation and extends beyond self-view ratings. Furthermore, across both studies, ISAs were positively correlated with alignment to normative ratings (Study 1: *r*_(4898)_= 0.19; Study 2: *r*_(5510)_= 0.52, both *Ps* < 0.001), suggesting that the reported self-view alignment with community norms may also be influenced by this artifact.

To further illustrate this point, we combined data from Studies 1 and 2 and tested whether ratings across different dimensions converge. Since people tend to rate positive traits as more self-descriptive (2), self-view, and valence ratings are typically positively correlated—even across individuals—leading to positive ISA. Indeed, our data showed a significant correlation between two ratings (mean *r* = 0.30, *P* < 0.001) and significant ISA (Fig. 2*C*), indicating that ISA can emerge even across dimensions.

Although the mean ISA values in our studies (see Fig. 2*C*) were smaller than those reported in the original study (mean = 6.77), this does not imply that the reduced noise bias is negligible. Several methodological differences exist between our studies and the original study. Notably, the short ~10-min gap [vs. at least a day (1)] between the first two ratings likely induced a stronger consistency bias in our participants, pulling ISA toward zero.

Our simulations and empirical studies clearly demonstrate that rating convergence across individuals can occur in the absence of any social interaction. While this does not necessarily invalidate the existence of the phenomenon, it does indicate that the original claims (including the findings on individual/dyadic differences) must be reevaluated with a proper control condition (see refs. 3 and 4 for a similar issue).
